# Invited Review: The role of prion‐like mechanisms in neurodegenerative diseases

**DOI:** 10.1111/nan.12592

**Published:** 2020-02-11

**Authors:** Z. Jaunmuktane, S. Brandner

**Affiliations:** ^1^ Division of Neuropathology National Hospital for Neurology and Neurosurgery University College London NHS Foundation Trust; ^2^ Department of Clinical and Movement Neurosciences and Queen Square Brain Bank for Neurological Disorders; ^3^ Department of Neurodegenerative disease Queen Square Institute of Neurology University College London London UK

**Keywords:** amyloid‐β, neurodegeneration, prion‐like, Tau, TDP43, α‐synuclein

## Abstract

The prototype of transmissible neurodegenerative proteinopathies is prion diseases, characterized by aggregation of abnormally folded conformers of the native prion protein. A wealth of mechanisms has been proposed to explain the conformational conversion from physiological protein into misfolded, pathological form, mode of toxicity, propagation from cell‐to‐cell and regional spread. There is increasing evidence that other neurodegenerative diseases, most notably Alzheimer’s disease (Aβ and tau), Parkinson’s disease (α‐synuclein), frontotemporal dementia (TDP43, tau or FUS) and motor neurone disease (TDP43), exhibit at least some of the misfolded prion protein properties. In this review, we will discuss to what extent each of the properties of misfolded prion protein is known to occur for Aβ, tau, α‐synuclein and TDP43, with particular focus on self‐propagation through seeding, conformational strains, selective cellular and regional vulnerability, stability and resistance to inactivation, oligomers, toxicity and summarize the most recent literature on transmissibility of neurodegenerative disorders.

## Introduction

**Figure 1:**
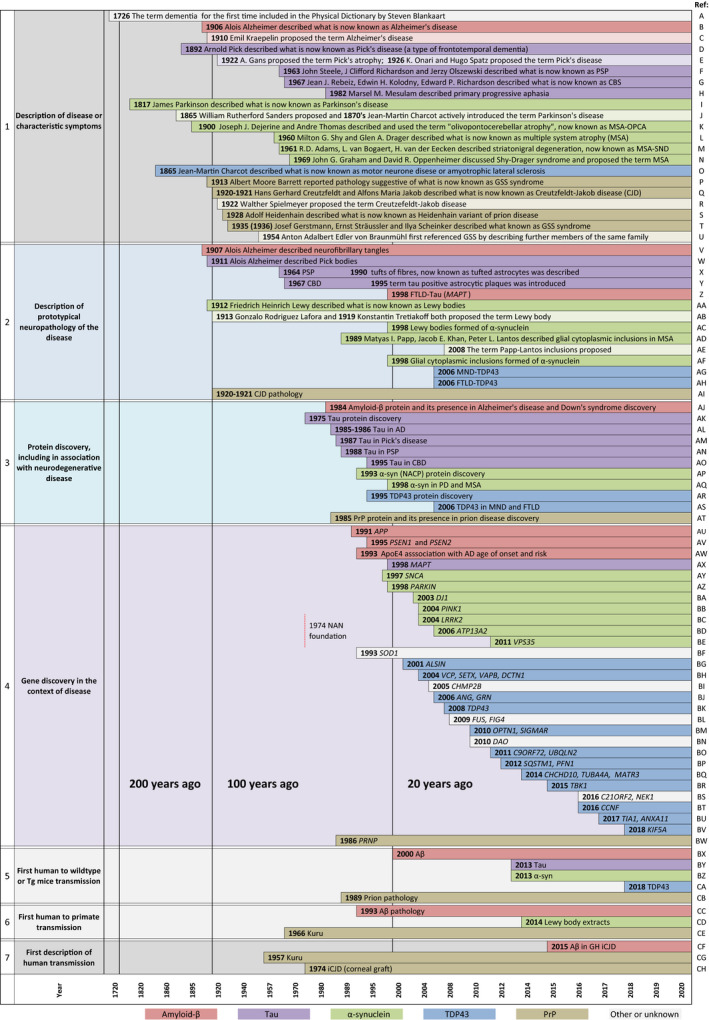
Timeline of landmark discoveries in neurodegenerative diseases. Graphical representation of the history of the discoveries and descriptions of diseases or symptoms, hallmark pathology, proteins, underlying genetic causes and first reported transmissions, as indicated in the left column. The timescale is represented as a Renard series to visualise recent discoveries in higher temporal resolution. Various discoveries related to different misfolded protein pathologies are represented colour‐coded, as indicated at the bottom of the figure. Row 1, first descriptions of the disease or symptom complexes, with separate representation of the time of the initial report and time of the introduction of the eponym, shown below in a corresponding lighter shade. Row 2 shows the timeline of the description of hallmark neuropathology for each disease. Row 3, the first discovery of the protein (for tau, α‐synuclein and TDP43 this precedes association of the misfolded protein with a specific disease). Row 4, the discovery of pathogenic mutations in various genes causing neurodegenerative diseases. The establishment of the journal Neuropathology Applied Neurobiology is displayed here to provide the context to other discoveries. Rows 5 and 6 display experimental transmission (from humans to rodents and primates, respectively) and row 7 indicates the first descriptions of human transmission. References: A 154; B 155, 156; C 156, 157; D 158; E 159, 160; F 161; G 162; H 163; I 164; J 165, 166; K 167; L 168; M 169; N 170; O 171, 172; P 173, 174; Q 175, 176; R 177, 178; S 179; T 170, 180, 181; U 174, 182; V 156, 183; W 184; X 185, 186; Y 185, 186; Z 187; AA 188; AB 189, 190, 191; AC 9, 10; AD 192; AE 193; AF 10; AG 11; AH 11; AI 175, 176; AJ 6; AK 194; AL 7, 195; AM 14; AN 13; AO 12; AP 196; AQ 10; AR 197; AS 11; AT 16; AU 198, 201; AV 202, 203; AW 204, 205; AX 187; AY 206; AZ 207; BA 208; BB 209; BC 210; BD 211; BE 212; BF 213; BG 214; BH 215, 216, 217, 218; BI 219; BJ 220, 221, 222; BK 223; BL 224, 225; BM 226, 227; BN 228; BO 229, 230, 231; BP 232, 233; BQ 224, 225, 226; BR 237; BS 238, 239; BT 240; BU 241, 242; BV 243; BW 244; BX 144; BY 59; BZ 117; CA 79; CB 245; CC 130; CD 126; CE 128; CF 41; CG 246; CH 137.

Protein aggregation diseases refer to a variety of disorders which develop as a result of the deposition of misfolded peptides or proteins in various organs, causing their dysfunction. The majority of adult neurodegenerative disorders is characterized by intra‐ or extracellular aggregation of misfolded proteins, leading to progressive neuronal and glial cell dysfunction and relentless progression of clinical symptoms. Over the last two centuries, there has been a continued growth of knowledge of the different phenotypes, pathologies and causes of neurodegenerative diseases (Figure 1).

Seminal studies on prion disease, undertaken mostly during the second half of the 20th century, have shown that the normal host prion protein (cellular PrP, PrP^C^) can undergo conformational change, and when misfolded (then referred to as PrP^Sc^) not only causes prion disease in its host but can also under certain circumstances be transmitted to the same or to other species. An essential prerequisite for such transmission and toxicity, discussed later in this review, is the presence of PrP^C^ in the recipient organism 1, 2. Over the last two decades, research into neurodegeneration has widened the scope, with a significant focus on investigating if and to what extent tau, α‐synuclein, Aβ peptide and more recently TDP43 exhibit similar properties. Stanley Prusiner, who received the Nobel Prize in 1997 for his work on prion disease, has always been a strong proponent that misfolded proteins associated with other neurodegenerative diseases may have similar properties. More than 30 years ago he hypothesized that experimental propagation of these proteins would require identification of a permissive host, appropriate conditions for replication and that transmission may manifest after very long incubation time 3. PrP^Sc^ displays characteristic properties, including (i) the formation of β‐sheet structures with a striking ability to form aggregates, often with protease resistance and with resistance to inactivation with conventional decontamination methods, (ii) seed formation with self‐propagation (i.e. an ability to induce abnormal conformation in a protein of the same kind, initiating a self‐amplifying cascade – termed template‐directed misfolding), (iii) the ability to form distinct conformations or strains, (iv) to propagate along neural pathways in the presence of host PrP^C^ 4, (v) exerting neurotoxicity and (vi) showing a selective regional vulnerability, which is modulated by strains and genetic polymorphisms, (vii) transmission within a species and across species, usually with a species barrier, and adaptation by serial passaging.

The term ‘prion’ was coined by Prusiner, with a loose reference to ‘proteinaceous infectious particle’ 5, describing an agent of transmissible spongiform encephalopathy with unconventional properties. This was intended to be an operational term that does not have structural implications other than that a protein is an essential component.

Subsequently, and particularly over the last decade, the term ‘prion‐like’ or ‘prionoid’ has been introduced into the literature to describe a misfolding and disease‐initiating mechanisms similar to, yet distinct from, prion disease. Numerous studies show that the proteins associated with neurodegenerative disorders such as Alzheimer’s disease (Aβ 6 and tau 7, 8), Parkinson’s and multiple system atrophy (α‐synuclein 9, 10), frontotemporal dementia (TDP43 11, tau 12, 13, 14 or FUS 15) and motor neurone disease (TDP43 11), exhibit at least some of the properties of abnormal prion protein 16 (the first reports of the misfolded protein presence in respective diseases are referenced here and also shown in Figure 1). However, the term ‘prion‐like’ is arguably one of the most overused terms in original studies and reviews published in the last 5 years and is applied with reference to different properties of misfolded prion protein or prion disease.

In an attempt to unriddle the controversies around the semantics, it has recently been proposed to define misfolded proteins which only partially display the properties of bona fide prions as ‘proteinaceous nucleating particles’ as opposed to the original definition of ‘prion’ 17. This is to emphasize that neurodegenerative disorders other than prion diseases may lack the same efficacy of transmission.

In this review, we will discuss to what extent each of the properties of prions is known to occur for Aβ, tau, α‐synuclein and TDP43.

## Amyloid structure

Amyloids are proteins with a propensity to aggregate into fibrils composed of cross‐β structures, which can be visualized with amyloid‐binding dyes, such as thioflavin S, thioflavin T or Congo red. Aβ, tau, α‐synuclein and, as shown also TDP43 18, birefringe under polarized light after Congo red staining, or fluoresce when stained with thioflavin S or thioflavin T. Instead, only FUS and SOD1 do not stain with amyloid dyes and therefore probably are not rich in β‐pleated sheets 18. Oligomers, the smaller assemblies of the protein fibrils, do not or only weakly react with amyloid dyes.

## Self‐propagation through seeding

The hypothesis of self‐replication of a protein in the absence of nucleic acids was first proposed in 1967 by the British scientists John Stanley Griffith, Tikvar Alper, Ian H Pattisson and Katharine M Jones, at the time working for the British government 19, 20. This hypothesis was later, in early 1980s, refined by the American neurologist Stanley Prusiner, who proposed that a protease‐resistant protein is a structural component of the scrapie agent 5.

The ability of misfolded proteins to seed, that is, to recruit physiological proteins of the same kind and to induce their conversion into a pathological form, and propagate from cell‐to‐cell with the continuous conversion of normal protein into misfolded form is the tenet of the protein‐only hypothesis. The ability of self‐propagation through seeding is the feature most frequently referred to when describing ‘prion‐like’ properties of other misfolded peptides and proteins. Growing experimental *in vitro* and *in vivo* evidence, indeed, indicates that templated corruption of like proteins is characteristic not only of PrP^Sc^ but also Aβ, tau, α‐synuclein (reviewed in 21) and TDP43 22. Furthermore, the ability of Aβ to enhance tau pathology, similar to what is speculated to occur in AD, has been demonstrated in several *in vitro* experiments, using tau seeding assays, and *in vivo* experiments, using transgenic and wild‐type mice 23, 24, 25, 26. Whether in AD Aβ, indeed, enhances tau pathology through mechanisms, such as neurotoxicity or proteostasis and clearance failure, or induces tau aggregation by direct cross‐seeding (or heterologous seeding), needs clarification through further research. Of note, in iatrogenic Creutzfeldt–Jakob disease (CJD) with concomitant Aβ pathology, it has been speculated that Aβ may have been induced by misfolded prion protein through cross‐seeding rather than iatrogenically transmitted. However, a recent experimental study does not support the cross‐seeding hypothesis 27.

## Strains (conformations) and seeding unit

The first description of strains (which derives from the early assumption of the causative agent of prion disease to be a virus) came from the observations in the early 1960s of distinct clinical phenotypes in goats, following experimental infection with scrapie brain extracts 28. These experiments also showed that the distinct clinical phenotypes were present even after several passages to other sheep.

There are at least four different human prion strains which are determined by distinct glycosylation characteristics of PrP^Sc^ and lead to a variation in the anatomical distribution of prion disease pathology, clinical symptoms, transmission properties, seeding efficiencies and *in vitro* amplification characteristics. It is now fairly established that the strain‐specific properties are encoded in the structure of the aggregated proteins and are retained when serially passaged *in vitro* or *in vivo* 29.

It has long been speculated that distinct structural conformations (strains) could also be a feature of other misfolded proteins and may, at least in part, explain distinct pathological and clinical phenotypes, and experimental transmission properties underlying AD, tauopathies, α‐synucleinopathies and TDP43 proteinopathies. Novel *in vitro* and *in vivo* models are constantly being developed to examine conformation, aggregation and propagation of misfolded proteins. These models are developed to specifically determine the minimal seeding unit required for initiating an efficient cascade of the seed amplification, propagation from cell‐to‐cell and spread from region‐to‐region. Recent reviews are available on the molecular basis and strain heterogeneity in neurodegeneration 30, the utility of bioassays to determine transmission 31 and cell free‐based methods for improved amplification and selective detection of PrP^Sc^ and other proteopathic seeds in biological materials, such as various tissues and fluids 32.

### Aβ strains and Alzheimer’s disease (AD)

Memory impairment is the most classic presentation of AD. However other initial symptoms, such as visual impairment characteristic of posterior cortical atrophy (PCA) clinical syndrome or behavioural changes and language impairment in the spectrum of frontotemporal dementia, are well known. It is tempting to speculate that this phenotypic diversity could be due to structurally distinct Aβ strains 33. Indeed, the existence of at least two distinct Aβ strains in AD has been shown in transmission studies in susceptible transgenic mice using sporadic AD and familial AD (FAD) brain homogenates 34. Another study, using solid‐state nuclear magnetic resonance, showed structural differences of Aβ species in patients with rapidly progressive, pathologically confirmed AD and those with PCA clinical phenotype 35. Conformational heterogeneity of Aβ42 in rapidly progressive AD has also been demonstrated using conformation‐dependent immunoassay and conformational stability assay 36. A further study, using luminescent conjugated oligothiophene binding to amyloid, showed differences in Aβ spectral properties in patients with sporadic AD and FAD 37.

The concept of the seeding unit is supported by *in vitro* data showing that intracellular oligomers can constitute such units and that seeded nucleation of Aβ can be intracellularly induced in an APP‐producing cell line when exposed to FAD brain extract 38. Using two transgenic mouse models (APP23 and APPPS1), it has been demonstrated that Aβ seeding potency is greatest during the earliest stages of Aβ deposition and coincides with a transient increase in the Aβ42/Aβ40 ratio; Aβ seeding potency decreases with increasing Aβ accumulation in the brain 39. This study highlights the potential importance of an early intervention with compounds that can interfere with Aβ seed formation well before amyloid deposits have aggregated to an extent to be detectable on imaging or *post mortem*. *In vivo*, Aβ oligomers in the CSF can be successfully detected with protein misfolding cyclic amplification assay (PMCA) 40.

Human transmission of Aβ pathology via medical procedures, such as contaminated cadaver‐derived pituitary hormone extracts, dura mater transplants and surgical procedures have been described by us and others 41, 42, 43, 44, 45, 46, 47, 48, 49. Transmitted Aβ pathologies cannot currently be distinguished histopathologically from sporadic or familial forms, unlike prion diseases, where, in some instances, iatrogenic transmission can be suspected on histological and biochemical grounds. This is possible in patients who are homozygous for methionine at codon 129 of the *PRNP* gene (129MM), but show a molecular prion strain type and kuru‐plaque pathology akin to prion disease patients with valine at codon 129 (i.e. 129MV or 129VV), suggestive of the pathogen originating from a patient with at least one *PRNP* 129V allele 50, 51.

### Tau

Tau deposition can be seen as primary pathology or as an accompanying secondary process in multiple neurodegenerative diseases. It is characterized by the predominance of either 3‐repeat (3R) tau, 4‐repeat (4R) tau or a mixture of 3R and 4R tau isoforms, generated by alternative splicing of exon 10 of the tau (*MAPT*) gene. The investigation, whether different tauopathies may be due to distinct tau conformers or strains, is an active area of research. Cellular and structural heterogeneity of tau conformations, as well as tau seeding, is demonstrated in several models *in vitro* and *in vivo* using nontransgenic and transgenic (P301S) mice and fluorescence resonance energy transfer‐based flow cytometry biosensor assays or Real‐Time Quaking Induced Conversion assay (RT‐QuIC) for tau seed detection 52, 53, 54, 55, 56. Tau substrates used in these experiments are of wide range including recombinant tau and preparations from human brains or CSF from patients with AD, chronic traumatic encephalopathy or Pick’s disease. Some research suggests that tau aggregation is induced by phosphorylated, high‐molecular weight tau fractions and not monomers 52, with the former also present in the CSF of an AD mouse model or AD patients 56. Recently, however, self‐assembly and aggregation properties have also been demonstrated *in vitro* for seed‐competent monomers extracted from AD brain tissue 57.

There is compelling experimental evidence that tau, similar to prions, stably propagates multiple conformations from different sporadic tauopathies *in vitro* 58 and induces distinct pathologies *in vivo* in mouse models reflecting the diversity of tauopathies in humans 55, 59, 60. Importantly, these tau conformations are transmissible and can be serially passaged *in vivo* and *in vitro* 58. However, there is also a key difference to prion diseases: mice expressing human P301L tau in the entorhinal cortex, crossed onto mice with tau‐null background show tau propagation and accumulation both in the presence and absence of endogenous tau, although lack of endogenous tau greatly reduces toxicity and brain atrophy 61. This finding indicates that, in contrast to prion protein, for tau transfer between neurones endogenous tau may not be obligatory. Further research is warranted to determine if distinct clinical presentations, disease progression dynamics and neuropathologies within a single disease entity [e.g. progressive supranuclear palsy (PSP)] can be explained by finer variations of the main tau strain or are determined by other factors, such as genetic, epigenetic or environmental influences.

Seeding activity *in vivo* has been established with insoluble tau aggregates in the form of short fibrils, but not small (>6mer) oligomeric tau assemblies 62. While in an *in vitro* study tau trimer is reported to be the smallest assembly that promotes dimerization of tau and could seed intracellular tau aggregation 63. This latter study, however, did not investigate tau trimer seeding and propagation ability *in vivo*, hence the smallest tau assembly capable to seed *in vivo* remains to be determined. The ability to seed tau from both soluble oligomers and insoluble tau fibrils suggests the existence of different tau seeds or conformers which may influence the speed of pathology as well as cellular response. Furthermore, the finding of widespread tau seeding activity in tissues containing both soluble and insoluble tau aggregates from brain regions preceding predicted Braak and Braak stages supports the hypothesis of tau spread along anatomically connected regions 64. Most seeding studies so far have used fresh or frozen tissue derived from human or experimental animal brains, but recently a protocol has been developed to extract and measure tau seeding activity from small volumes (0.04 mm^3^) of formaldehyde‐fixed tissue, which may enable successful studies of tau seeding activity in diverse tauopathies using archival formalin‐fixed brain tissues 65.

### α‐synuclein

There are three diseases characterized by pathological α‐synuclein deposits, affecting neurones and glial cells. Dementia with Lewy bodies (DLB) and Parkinson’s disease (PD) show predominantly neuronal Lewy body pathology, and for the third, multiple system atrophy (MSA), hallmark pathologies are glial cytoplasmic, and to a lesser extent, neuronal inclusions. Both PD and DLB show similar pathology, with differences in clinical phenotypes. MSA pathology can present with several distinct clinical phenotypes with variations of the pathology burden in affected anatomical regions.

Both *in vivo* and *in vitro* experiments indicate that brain homogenates from patients with MSA, but not with PD, induce neurodegeneration in transgenic mice (TgM83+/‐) and in cell models (α‐syn*A53T‐YFP) 66. Furthermore, an experiment involving serial propagation of MSA α‐synuclein between two different mouse models showed retained specificity of pathological conformers 67.

Recently, further differences in seed characteristics and seeding activity have been demonstrated between soluble and insoluble fractions of PD and MSA extracts, which persist in second‐generation biosensor cells 68. In this study, MSA extracts showed seeding activity for soluble and insoluble fractions, while for PD extracts it was only the insoluble fraction which displayed seeding activity 68. Findings from a study specifically looking into the seeding activity of pathological α‐synuclein extracted from DLB patients, measured with RT‐QuIC, suggests that in extracts from DLB patient brains prefibrillar soluble α‐synuclein oligomers, and not insoluble forms, exert seeding activity 69. Structural differences in the seeding kinetics using RT‐QuIC have been also observed when comparing DLB and PD cases 70. Experiments like these, support at least indirectly the notion that MSA, PD and DLB, are caused by different α‐synuclein strains. RT‐QuIC is a sensitive and specific method for misfolded prion protein detection 71 and it can also detect with high sensitivity and specificity pathological α‐synuclein in both, brain homogenates and, more importantly, CSF from patients with PD and DLB 72, 73. α‐synuclein seeding activity, using specially optimized PMCA protocol, has been demonstrated in formalin‐fixed MSA brain tissue 74.

### TDP43

The majority of motor neurone disease (MND) and all FTLD‐TDP43 cases are caused by aggregated phosphorylated TDP43. There are five subtypes of FTLD‐TDP43, designated types A‐E, which differ in terms of the shapes of pathological aggregates, affected brain regions and clinical phenotypes. TDP43 pathology is also seen in elderly patients in the context of certain other neurodegenerative diseases (e.g. PSP and AD), or as part of the ageing process. As with other neurodegenerative diseases, it has been hypothesized that the distinct FTLD‐TDP43 types may be due to different TDP43 conformations, but this is currently not yet underpinned by strong experimental evidence. However, biochemical differences between phosphorylated TDP43 (pTDP43) extracted from distinct FTLD‐TDP43 types and differences in their abilities to interfere with neurite growth have been recently shown 75. Formation of oligomers during the early stages of TDP43 misfolding 76, 77 and differences in staining properties with TDP43 oligomer‐specific antibodies has also recently been demonstrated for distinct FTLD‐TDP43 types and in patients with hippocampal sclerosis with TDP43 pathology 78. In a cell‐based seeding assay, using inducible GFP‐tagged cytoplasmic TDP43, extracts derived from brains with sporadic and familial FTLD‐TDP43, indeed, show more seeding than control brain extracts, with the highest seeding activity seen in samples with *GRN* mutation, while *C9ORF72* mutant and sporadic cases show no difference 79. All these experiments can be regarded as circumstantial evidence of distinct TDP43 strains.

TDP43 consists of three domains, an N‐terminal Dix‐like domain mediating self‐assembly, two RNA recognition motifs, and an intrinsically disordered low complexity domain (LCD) at the C terminus. Majority of TDP43 inclusions contain C‐terminal fragments of LCD, which have a tendency to form amyloid fibrils 80. TDP43 aggregation, at least in part, is governed by a process called liquid–liquid phase separation (LLPS), which is a reversible process, characterized by fluid de‐mixing into distinct liquid phases. LLPS is increasingly recognized as a possible source of protein self‐assembly into larger aggregates. In neurodegeneration, this has been most studied concerning RNA‐binding proteins TDP43 and FUS, although LLPS also occurs in tau 81 and prion protein 82. Recent studies show that for TDP43 to undergo LLPS as few as three tryptophan residues are required 83, however, minimum seeding unit required for efficient induction of TDP43 pathology is not yet ascertained.

## Selective regional vulnerability

A characteristic spatiotemporal distribution of Lewy bodies in PD, Aβ and tau in AD and grain pathology in argyrophilic grain disease is typically seen during disease progression. This progression extends across neuro‐anatomically connected regions (Lewy body pathology and neurofibrillary tangle pathology) or to some degree in relation to spatial proximity (Aβ 84). These observations have formed the basis of various pathological staging schemes 85, 86, 87. Similar patterns of spread may also exist for other α‐synucleinopathies, tauopathies and TDP43 proteinopathies, and for some (notably secondary TDP43 in AD), such staging schemes are suggested 88, 89.

Prion diseases are a prime example where selective regional vulnerability is associated with clinical symptoms and syndromes, and genetic modifiers of the pathology and phenotypes have been well characterized. For example, inherited prion disease due to D178N mutation in the *PRNP* gene can cause two distinct clinical phenotypes and pathologies. These phenotypes are determined by the methionine (M) and valine (V) polymorphism on the codon 129 of the *PRNP* gene. Coupling of the D178N mutated codon with codon 129M on the same allele results in fatal familial insomnia with the main pathology in the thalamus, while CJD phenotype with dominant cortical pathology requires coupling of the mutated codon with codon 129V.

Such striking correlate has not yet been established for any other neurodegenerative disease, although selective regional vulnerability is a feature of most, leading to differences in clinical phenotypes. For instance, the same mutation in the *MAPT* gene can show marked phenotypic and pathological diversity, but factors contributing to this heterogeneity remain to be determined 90. In fact, the *MAPT* A152T mutation may confer risk to completely different diseases, such as AD, corticobasal degeneration (CBD), PSP and DLB.

Of note, phenotypic heterogeneity in inherited prion disease due to P102L mutation in the *PRNP* gene, in part, is explained by variable involvement of wild‐type PrP^Sc^ 91. By drawing further parallels with inherited prion disease, one explanation for phenotypic variability in inherited tauopathies, TDP43 proteinopathies and AD, could be the extent of non‐mutant protein misfolding and propagation.

**Figure 2:**
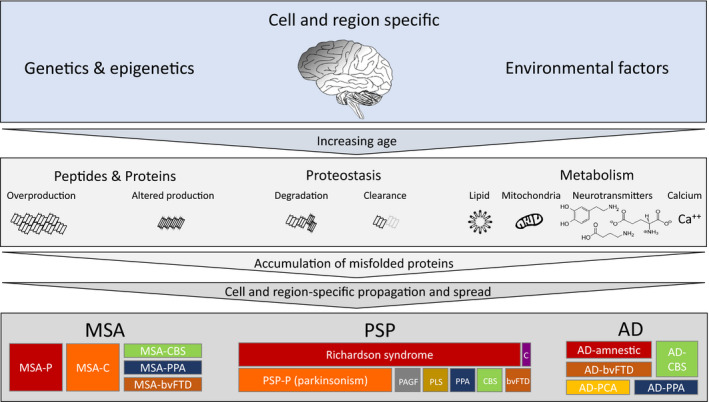
Determinants of phenotypic diversity of neurodegenerative diseases. Cell‐ and region‐specific genetic, epigenetic and environmental factors with increasing age play an essential role in neurodegeneration development, highlighted in the top row. The central panel illustrates the most significantly involved or best characterised subcellular processes, which lead to the initiation of pathological protein aggregation. These include overproduction or abnormal production of specific peptides and proteins, disturbance in the proteostasis with impaired degradation of misfolded proteins or reduced clearance through various fluids and compromised metabolism (mitochondria, lipid metabolism, neurotransmitter, such as glutamate, dopamine or GABA, and calcium homeostasis). Impairment of one or a combination of the above, leads to initiation of misfolded protein aggregation and self‐amplification, with a cell‐specific propagation and region‐specific spread, resulting in distinct clinical phenotypes, represented in the bottom panel. Multiple system atrophy (MSA), α‐synucleinopathy with its hallmark glial cytoplasmic inclusion pathology, shown on the left side, typically presents with parkinsonism (MSA‐P) or cerebellar signs (MSA‐C). However rare clinical presentations, such as corticobasal syndrome (MSA‐CBS), primary progressive aphasia (MSA‐PPA), and behavioural variant frontotemporal dementia (MSA‐bvFTD) are well known. Progressive supranuclear palsy (PSP), a primary tauopathy with characteristic tufted astrocyte pathology, shown in the centre of the panel. In addition to the most widely known Richardson syndrome (PSP‐RS), PSP can have other clinical presentations, such as PSP‐P which closely resembles parkinsonism; pure akinesia with gait freezing (PSP‐PAGF), primary lateral sclerosis dominated by corticospinal tract degeneration (PSP‐PLS), primary progressive aphasia (PSP‐PPA), corticobasal syndrome (PSP‐CBS), behavioural variant frontotemporal dementia (PSP‐bvFTD) or the rarest presentation dominated by cerebellar signs (PSP‐C). Also for Alzheimer’s disease, in addition to the most common amnestic presentation, other clinical phenotypes include behavioural variant frontotemporal dementia (bvFTD), posterior cortical atrophy (AD‐PCA), corticobasal syndrome (AD‐CBS) and primary progressive aphasia (AD‐PPA).

Tauopathies are among the most striking examples of heterogeneity of affected cell types and anatomical regions, resulting in distinct clinical phenotypes (Figure 2). Even within 4R tauopathies, there is a great variation in tau pathology, atrophy and clinical presentation. For example, there are eight known clinical phenotypes of PSP, each with overlapping but also distinct anatomical involvement. Current experimental research suggests the existence of different pathological tau strains in AD, PSP and CBD, which maintain cell‐type specificity in nontransgenic mice 55. Hence, selective cellular vulnerability in different tauopathies may, at least in part, be strain‐related. However, the same study, by injecting comparable concentrations of tau purified from AD, CBD and PSP brains, into the brains of nontransgenic mice, showed similar neuroanatomical distribution of tau pathology, albeit with differences in density between different diseases or the same disease, but with distinct phenotypes. Similar neuroanatomical distribution of transmitted tau pathology from different tauopathies suggests that selective regional anatomical vulnerability is not directly strain related. However, the observed differences of tau pathology burden using different human brain samples with the same disease, indicates the existence of variably potent tau strains, resulting in variably severe regional burden of tau pathology and diverse clinical phenotypes of the same disease (Figure 2).

Large gaps remain in the understanding of the pathobiology of selective regional vulnerability in neurodegeneration. Increasingly it is recognized that complex and diverse genome‐wide alterations, gene expression patterns and epigenome‐wide DNA methylation profiles play a role in cellular and anatomical vulnerability in neurodegeneration. They modify neuronal, glial, and microglial cell function, clearance and degradation of misfolded proteins, factors influencing spread, such as exocytosis and cellular uptake, or cause alterations in signalling pathways. Furthermore, genetic and epigenetic factors also modulate resistance to harmful proteopathic seeds and eventually much of the phenotypic and pathological variability is probably caused by a combination of the above (Figure 2). A recent systematic genome‐wide review discusses common molecular pathways involved in neurodegeneration and highlights the complexity of involved pathways spanning from protein degradation, mitochondrial function, inflammatory response, metabolism of lipids and cholesterol 92.

Of note, recent research suggests that at least for PD, the genetic risk loci do not lie in specific brain regions or restricted (e.g. solely neuronal or glial) cell types, but, instead, they are present across a range of cell types with involvement of a number of global pathways 93. These observations highlight the need for further research to better understand the interplay between cell and region specific (epi)‐genetic alterations and selective cellular and regional vulnerability in various neurodegenerative diseases.

## Stability and resistance to inactivation

One of the defining features of PrP^Sc^ is the relative resistance to proteolysis by proteases. This was discovered in the 1930s in the context of vaccine development against louping‐ill virus, a devastating endemic tick‐borne illness in sheep. The virus was isolated from spleens and brains of affected sheep, and inactivated with formalin. However, scrapie agent, unknowingly present in the starting material, was transmitted into vaccinated sheep who succumbed to typical scrapie 94. More recently, it has been shown that also Aβ from AD brains and α‐synuclein from MSA brains is not inactivated by formalin; and formalin‐fixed human brain tissue extracts from patients with AD and MSA or transgenic mice with respective pathologies have been transmitted to susceptible transgenic mice where they elicit corresponding pathology 95, 96, 97. Such stability is also relevant for the decontamination of surgical instruments. PrP^Sc^, Aβ and α‐synuclein adhere to steel and show remarkable resistance to ‘inactivation’ with standard decontamination procedures and elicit prion disease, Aβ pathology 98 and MSA 96 pathology respectively. Instead, the transmission of tau, TDP43 or Lewy body pathologies from formalin‐fixed tissues or through steel wires has not been reported to date. It is also worthwhile to note, that studies showing transmission of tau, TDP43 and Lewy body pathology after intracerebral injections in various experimental models have utilized tissue enriched in pathology with excessive quantities of the pathological conformers. Furthermore, it is curious, that differences in proteinase K proteolytic properties are documented in DLB and PD patient brain extracts subjected to RT‐QuIC analysis, with amplified α‐synuclein from DLB, but not PD or control cases, showing proteinase K resistant species 70.

## Oligomers and toxicity

The term ‘toxicity’ may imply a number of different effects at biochemical and molecular level involving cells and their networks. This also means different measurements of the toxicity, which may vary, for example, from the assessment of cognition with an array of behaviour tests, or assessment of the synaptic or mitochondrial function in tissues using neurophysiological and biochemical assays 99. Seeded templating and neurotoxicity are two of the most critical properties attributed to oligomers. Oligomeric states have been documented for misfolded proteins involved in neurodegeneration, that is, prion protein, Aβ, tau, α‐synuclein, huntingtin, TDP43, mutant nuclear (but not cytoplasmic) FUS 100 and mutant SOD1 in MND 101.

A growing body of *in vivo* data suggests that soluble oligomers of PrP^Sc^ 102, Aβ 103, tau 99, α‐synuclein 104 and TDP43 77 are among the key components to induce seeding and cause neurotoxicity with neurodegeneration. It has been proposed that the efficiency of self‐amplification occurs at oligomer concentrations that are different from those causing toxicity, as it has been demonstrated for α‐synuclein 105. For Aβ, specifically the longer forms (1–42 and 1–46) show greatest synaptotoxicity, and synaptic function can be restored upon prevention of soluble Aβ formation and/or treatment with antibodies targeting specifically N‐terminal residues 103.

Differences in neurotoxicity and seeding activity have recently also been established in FTLD‐TDP43 subtypes. pTDP43 extracts from FTLD‐TDP43 type A show the most severe toxicity with a reduction in neurite length 75 and similar findings are described for *in vitro* generated α‐synuclein oligomers 106. Yet, it is not established if (or how) oligomers initiate neurodegeneration or if neurodegeneration could be merely the consequence of misfolded protein aggregation intra‐ and extracellularly, separate from propagation and toxicity.

A number of key questions related to oligomers, toxicity and propagation remain to be answered: (i) are oligomers responsible for cell‐to‐cell propagation across specified networks or defined anatomical pathways; (ii) to what extent aggregation and propagation of oligomers, protofibrils and larger fibrils corresponds to toxicity, and if therapeutic attempts to inhibit oligomer formation or fibril aggregation and propagation can eliminate neurotoxicity; (ii) are neurotoxic oligomers identical to the transmissible agent; (iii) is toxicity disruptive to cells or networks at a biochemical level (such as neurotransmitter transport, cytoskeletal, organelle and synaptic integrity), and to what extent does it cause cell loss and brain atrophy; (iv) what specifically is the cause of oligomer abundance – for example, overproduction, clearance failure (extracellular pathways, such as blood–brain barrier or glymphatic system), or loss of proteostatic integrity within lysosomes, autophagosomes, proteasomes, ubiquitin‐peroxisomal system or any other less understood pathways for misfolded protein degradation and/or excretion out of the cell (Figure 2); (v) do different oligomer sizes (i.e. small oligomers in the range of 2–5 mers (repeat units) and large oligomers (in the range of 6–150 mers) and concentrations have distinct roles in each proteinopathy; (vi) do soluble and structure (such as vesicle) – bound oligomers have distinct roles; (vii) are the current methodological approaches used for oligomer detection and quantification sufficient and what are the challenges for the development of a reproducible and most accurate measurement of oligomer size and concentration in tissues and fluids.

## Propagation and transmission

The commonly used term ‘prion‐like property’ describes the ability of aggregated, misfolded proteins, other than prion protein, to act as a template for the formation of seeds from a physiologic form of the same protein, propagate between cells and spread across anatomical pathways. The use of the term ‘prion‐like spread’ has provoked controversy, as it presumes a unifying mechanism of the propagation of misfolded proteins in the nervous system and oversimplifies the complexity of the pathomechanisms underlying the spread of different proteopathic seeds and neurodegeneration development.

**Figure 3:**
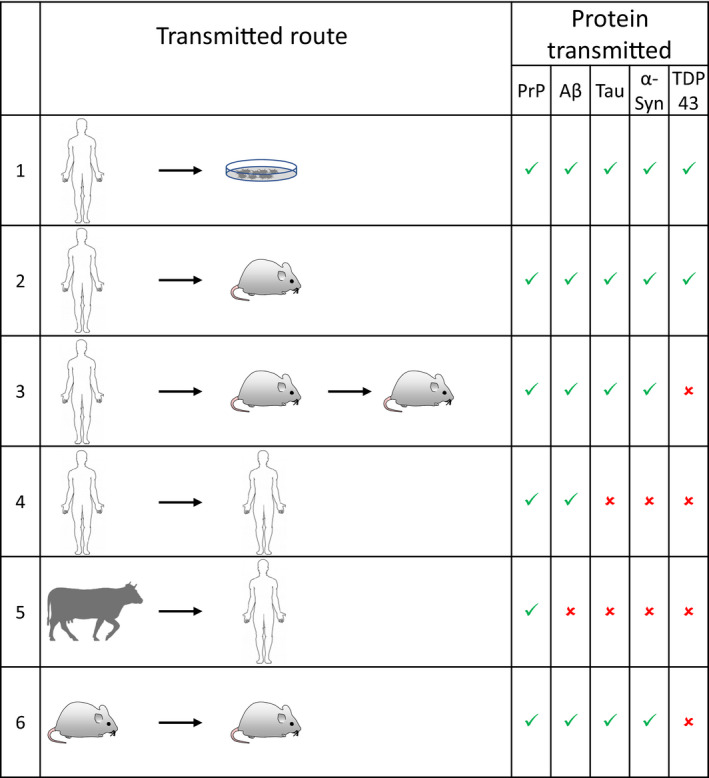
Transmission routes of proteopathic seeds. Rows indicate the demonstrated transmitted route and the transmitted peptides or proteins are shown in the columns on the right. Green ticks indicate evidence of successful transmission and red crosses indicates absence of evidence of successful transmission. Rows 1, 2; transmission into cells and animal models has been accomplished for all 5 proteins. Row 3, serial transmission into animals has been accomplished for PrP, amyloid‐β and α‐synuclein, but so far has not been shown for TDP43; for α‐synuclein and tau conformers this has not been demonstrated for all human diseases. Row 4, transmission between humans is known to occur for PrP and amyloid‐β pathologies, for tau, α‐synuclein or TDP43, this has not been demonstrated and further studies are required. Row 5, transmission from animal species into humans has to date only been reported for prions (bovine spongiform encephalopathy and variant CJD). Row 6, experimental transmission from mice (usually genetically modified) into the same species, has been reported for prions, amyloid‐β, tau, and α‐synuclein.

An important obvious difference between PrP, Aβ, tau, α‐synuclein and TDP43 is the transmembranous and extracellular location of PrP and Aβ, while tau, α‐synuclein and TDP43 are predominantly intracellular. Therefore, arguably, only the propagation of Aβ peptide (and of ADan and ABri proteins, found in exceptionally rare familial dementias), could be likened to a prion‐like spread, as, similar to prions, these are located in the membrane and extracellularly. Experimental paradigms to test seeding activity include cell models to demonstrate propagation *in vitro* and animal models to show transmissibility. Another scenario is where transmission occurs between individual organisms within species (such as scrapie and bovine spongiform encephalopathy) or, theoretically, across different species. The following paragraphs will highlight the characteristics of *in vivo* propagation within an organism (propagation between cells) or scenarios where transmission occurs between individual organisms of the same or different species. Figure 3 summaries the confirmed transmission routes of various proteinopathies.

## *In vivo* propagation between cells

The first observation of cell‐to‐cell transmission in the human brain dates back to 2008 when two groups reported Lewy body pathology development within foetal dopaminergic neurone grafts, which had been implanted in basal ganglia of PD patients many years earlier 107, 108. This ground‐breaking observation was followed by many *in vitro* and *in vivo* experiments designed to better understand the mechanism of cell‐to‐cell spread of proteopathic seeds. Many physiological pathways, such as trans‐synaptic and non‐synaptic (interstitial diffusion, micro‐ or macro‐pinocytosis, exosomes, vesicle‐mediated exocytosis–endocytosis or nanotubes 21) are candidates for transcellular spread. While it is beyond the scope of this review to discuss all these pathways in detail, it should be mentioned that there are cell culture models for tau, α‐synuclein, TDP43 22, 109 and more recently also intracellular Aβ seeding and nucleation 38. Also, the role of astrocytes and microglia for efficient trans‐neuronal propagation is increasingly acknowledged. For example, microglial activation in embryonal dopaminergic neurone grafts has recently been shown to be present from early on after graft implantation and well before Lewy body pathology development in these grafts 110. Such observations underpin the complexity of factors that can contribute to the initiation and spread of proteopathic seeds.

## Transmission of proteopathic seeds *in vivo*

### The transmission between animals of the same species

The transmission between animals of the same species was first described in prion disease in 1936–1939, when scrapie transmission to sheep, by intraocular injection, was demonstrated by two French veterinary scientists 111, 112, 113. Over subsequent decades many experimental *in vivo* models have been developed with the overarching aim to investigate the mechanisms of transmission and propagation and seeding potencies of misfolded proteins implicated in neurodegeneration. Aβ transmission in transgenic mice was first reported in 2000 114, mutant P301S tau transmission in transgenic mice in 2009 115 and from human tauopathies into transgenic mice in 2013 59; α‐synuclein transmission in transgenic mice using synthetic fibrils in 2012 116 and human MSA brain extracts in 2013 117, and TDP43 in transgenic mice using human brain‐derived FTLD‐TDP43 extracts in 2018 79. Most common experimental approaches include intracerebral injections of brain extracts derived from transgenic mice or humans into transgenic mice expressing the human form of the protein or into wild‐type mice. For Aβ and tau, intracerebral propagation of respective pathology can also be seen after extended incubation times, following peripheral administration of Aβ 118 and tau aggregates 119 derived from transgenic mouse models either overexpressing APP (APP23) or expressing mutant tau (P301S) respectively. Similarly, neurological impairment and α‐synuclein pathology are demonstrated to develop in the CNS of transgenic mice following intramuscular administration of α‐synuclein fibrils 120. Recently, α‐synuclein transmission in transgenic mice has been demonstrated following oral, intravenous and intraperitoneal administration of recombinant α‐synuclein fibrils, made of human wild‐type α‐synuclein 121. 

### Experimental transmission between animal species

Experimental transmission between animal species can be useful to determine the species barrier and, thus, the zoonotic potential. Cross‐species transmission of prions has been extensively studied. Scrapie transmission was demonstrated for the first time in goats in 1959 122, and soon after into wild‐type mice 123. The most prominent zoonosis is Bovine Spongiform Encephalopathy (BSE), but several other highly prevalent prion diseases in animals exist with unascertained zoonotic potential. BSE cross‐species transmission was first proven in wild‐type mice (1988) and marmosets (1993) followed by transgenic mice, sheep, macaque and vole. A comprehensive review of transmission studies of prion diseases is in 124. Recently, scrapie transmission after prolonged incubation periods has been demonstrated also in primate, cynomolgus macaque, which show high PrP protein homology to humans 125. Apart from prion disease, there is currently no evidence that other proteins involved in human neurodegeneration can be transmitted between animals. A strong research focus instead is on studying the potential of experimental transmission of diseased human brain tissue into animal models.

### Transmission from humans to animals

Transmission from humans to animals is exclusively seen in experimental settings. In transgenic and wild‐type mice, cell‐to‐cell propagation has been demonstrated with intracerebrally injected human brain extracts from AD, various primary tauopathies 59, α‐ synucleinopathy (PD 126 and MSA 117), and more recently also from patients with FTLD‐TDP43 79. The first transmission of a human proteinopathy was prion disease *kuru* into chimpanzees in 1966 127, 128. More than two decades later, the transmission of Aβ pathology (CAA and parenchymal deposits) 129, 130 and recently, also α‐synuclein Lewy body pathology transmission was accomplished in primates 126. Instead, the transmission of tau and TDP43 pathology into primates has yet to be confirmed.

### Transmission of human misfolded proteins into cell models to study in vitr*o*

Transmission of human misfolded proteins into cell models to study *in vitro* propagation of neurodegenerative disease is an active and extensive research area. These studies are essential to study specific aspects of cell biology and biochemistry of these proteins. As of now, all major neurodegeneration‐related misfolded proteins have been propagated in various cell models (Figure 3) 131, 132, 133, 134, 135.

### Transmission between humans

Transmission between humans was first described in prion diseases: kuru was a major epidemic of human prion disease in the people of the Fore linguistic group in the Eastern Highlands of Papua New Guinea. It was transmitted through the practice of engaging in the consumption of dead relatives as a mark of respect and mourning (transumption) 136. Iatrogenic transmission of prion disease was first reported in 1974 in a patient who had received a cadaveric corneal transplant from a donor who, in retrospect, had been identified as having died of CJD 137. Subsequently, the transmission of CJD through cadaver‐derived hormones, most prominently growth hormone (GH), and contaminated dural transplants during neurosurgical (and surgical) procedures have been reported. This resulted in a ban on the use of human cadaver‐sourced hormones in 1985 and cadaver‐derived dural transplants in the 1990s.

**Figure 4:**
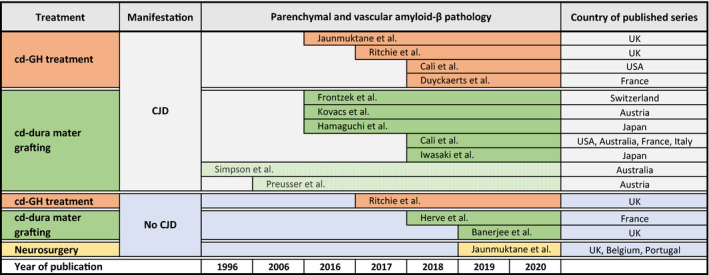
Recent history of published observations on human transmission of amyloid‐β. Initially, the human transmission of amyloid‐β through iatrogenic procedures was reported in the context of transmitted prion disease, with the first landmark study in 2015 documenting widespread parenchymal and vascular amyloid‐β pathology in patients who had received human cadaver derived GH (cd‐GH) treatment several decades earlier. This was followed by a similar observation in patients who had received cadaver derived (cd) dura mater treatment. Later studies described amyloid‐β transmission also independent from prion disease, so far through cadaver derived growth hormone treatment, dura mater grafting and neurosurgical instruments. Two case reports, shown in light green, refer to earlier reports describing amyloid‐β pathology, but not suggesting potential iatrogenic transmissibility. On the far right, the countries of published series are specified.

For many decades, it was thought that misfolded proteins other than abnormal prion protein do not transmit between humans. This assumption was based on the lack of epidemiological evidence and the absence of any overt clinical neurological manifestations in primate studies 138. Over the last 5 years, this view has fundamentally changed since the publication of several studies providing circumstantial evidence of human transmission of Aβ. In 2015, we observed that patients, who had received pituitary‐derived GH and had died of prion disease, also showed frequent cerebral amyloid angiopathy (CAA) and parenchymal Aβ 41. After this landmark publication, Aβ transmission was also demonstrated in patients who had received contaminated dural grafts 44. Since then, many authors have reproduced these findings in various cohorts of iatrogenic CJD patients 43, 45, 46, 47, 139. In retrospect, Aβ pathology in dural graft‐related iatrogenic CJD patients had already been reported before 140, 141, albeit without association with potential transmissibility. Patients who had not contracted CJD, but died from other causes also show Aβ pathology 43. Furthermore, some have developed complications related to amyloid angiopathy, such as intracerebral haemorrhages 48, 49. The pathogenic role of Aβ seeds has been confirmed by the transmission of pituitary‐derived GH, archived in vials for decades, into transgenic mice that developed accelerated amyloid angiopathy 142. A further mode of Aβ transmission was uncovered when we examined unexplained early‐onset CAA in a small series of patients in the 3rd and 4th decades of life. When exploring the clinical history we found that these patients had neurosurgical interventions during childhood with no evidence of cadaveric dural grafting 42, suggesting Aβ transmission through neurosurgery 42 (Figure 4). To date, CAA‐related haemorrhages have been the main complication of iatrogenically transmitted Aβ 42, 48, 49, prompting public health concerns. Cases reported to date have not shown evidence of advanced AD pathology.

Human transmission of tau, TDP43 and α‐synuclein pathology has not been proven as yet, but it may just be a matter of time until epidemiological, pathology‐confirmed studies have been conducted. Review of clinical notes from patients, who had died of MSA and PD, showed no evidence of neurosurgical transmission or cadaver‐derived GH treatment 143 and a study of five couples whose spouses had pathologically verified PD, PSP or MSA also did not suggest an increased risk of α‐synucleinopathy development in the other spouse 144. Current absence of evidence, however, is not evidence of the absence of human transmission of misfolded proteins other than prion and Aβ, and further research is necessary before any firm conclusions can be drawn.

A possible explanation of the transmissibility of PrP^Sc^ and Aβ, but not tau, α‐synuclein and TDP43, may be related to the differences in cellular localization (extracellular and transmembrane *vs.* cytoplasmic), rendering prion protein and Aβ more readily available for iatrogenic seed transmission. Another explanation could be related to the incubation periods, which for prion diseases vary from a couple of years to over 40 years 51 and for Aβ, as the current observational studies show (Figure 4), at least 20 years are needed for the pathology to be detectable and manifest clinically. It is plausible that even longer incubation periods, possibly exceeding human lifespan, are required for tau, TDP43 and α‐synuclein pathology development. Further differences could be due to critical mass of initial seeds required, to initiate a self‐amplifying cascade. For example, *in vitro* studies show that while for tau the minimal propagation unit is as small as tau trimers, larger oligomers comprising up to 100 tau molecules have the greatest seeding efficiently 63. A much higher concentration (at least 10,000 fibrils or oligomers per cell‐like volume) of α‐synuclein, is needed for efficient seeding, with fibrils being more effective at seeding than oligomers 105. In contrast, for tau, using a similar methodological approach (single‐molecular fluorescence resonance energy transfer and kinetic analysis), significantly less oligomers appear to be required for efficient seeding 145. In comparison, in sporadic prion disease in patients with MM genotype at codon 129 of the *PRNP* gene, the average 50% seeding dose (SD), assessed with RT‐QuIC assay, corresponds to approximately 10^10^ /g brain, or 1 SD_50_ unit equivalent to 0.06–0.27 fg of PrP^Sc^ 146. As demonstrated in these examples, different methodologies for different neurodegenerative diseases have been applied, with some measuring the oligomer size or number of molecules, and others estimating the concentration or mass.

Of note, research applying kinetic analysis also allow to predict most effective conditions for templated seeding to occur and the suggested relevant factors are: nucleation rate (small number of oligomers or fibrils show most effective seeding at slow nucleation rate) and initial protein concentration and its relationship to critical aggregation concentration (templated seeding is more effective at low concentrations with fewer oligomers needed) 105.

### Transmission from animals to humans

Transmission from animals to humans has probably only occurred in the context of BSE. Owing to changes in processing cattle feed, BSE first spread endemically in cattle and was then transmitted, most likely through the food chain, to humans 147. It caused an unusual, early‐onset neurological syndrome, which was later designated as variant CJD (vCJD). Polymorphic variants of the *PRNP* gene in humans and animals have a strong influence on the transmissibility and development of a clinical phenotype. All but one cases of vCJD occurred in patients with the *PRNP* codon 129MM genotype. The exception is a patient who presented clinically and radiologically with a sporadic CJD phenotype but showed histologically a prototypical vCJD, and biochemical typing confirmed the BSE/vCJD strain 148. For last several years, no new cases of vCJD have emerged, however, it is debated if the vCJD strain could have adapted into a clinically and histologically classical CJD 149, making it nearly impossible to identify as transmitted form.

Discovery of unusual forms of misfolded protein diseases, such as parkinsonism‐dementia complex of Guam and the recent report of a tauopathy in young East‐African children with nodding syndrome 150, make one consider the possibility of environmental causes, leading to cross‐species transmission of proteins other than prion. As always, such speculations need to be viewed with extreme caution. For tau pathology in particular, its development in patients with long‐standing seizures is well known 151. Thus, tauopathy that has been described in the nodding syndrome, a form of epilepsy with uncontrollable nodding of the head, may well be secondary to the seizures. Recent research, in fact, suggests that epilepsy in patients with nodding syndrome is an immune‐mediated reaction related to parasitic worm onchocerciasis infection 152, further suggesting that tauopathy may, indeed, be secondary.

## Conclusion and future perspectives

The concept of a transmissible disease, caused by seeds or template‐directed self‐assembly of proteins has fascinated the scientific community for decades. As highlighted in this review, over the last two decades building on century‐long research in prion diseases, startling revelations, both practical and profound, have been made. The research has furthered the understanding of mechanisms of proteopathic seed development, templated seeding, propagation from cell‐to‐cell and spread from region‐to‐region. While likening misfolded proteins and peptides of common neurodegenerative diseases, such as AD and PD, to prion disease, may have some value in translating the research findings from one field to the other, such comparisons are not always accurate or used attentively. Large gaps remain in understanding the mechanisms of prion and all other neurodegenerative diseases.

The future research focus should be twofold: Firstly, related to public health, to ensure adequate surgical and laboratory instrument decontamination procedures are in place, and prospective epidemiological studies are designed to specifically investigate any potential transmissibility of misfolded proteins and peptides which require long incubation periods to be detectable. There are defined requirements for biosafety procedures to handle cell and animal models, and human tissues in prion disease research and clinical practice. Instead, there are currently no data suggesting that such precautions are warranted for other protein species. Any recommendations to introduce similar requirements for research into other neurodegenerative diseases would add substantial financial and operational barriers and would obstruct advancements in the field. Adherence to good laboratory practice and health and safety guidelines, relevant to human and experimental animal tissue, is currently considered to be appropriate for handling material from neurodegenerative disorders other than prion diseases (see also review: 153). In clinical practice, patients at risk of developing iatrogenic Aβ pathology, notably CAA, should be considered to be followed up with neuroimaging and with appropriate CSF biomarker tests, such as Aβ, tau and other neurodegenerative proteins (e.g. neurofilament light chain). Secondly, further experimental research is needed to unravel the molecular and mechanistic processes leading to initiation of self‐amplification, efficient propagation and spread of proteopathic seeds. The exact role of oligomers in causing neurotoxicity, regional vulnerability, transmissibility and neurodegeneration as discussed in this review needs to be determined. The complexity of genetic and epigenetic alterations, including the role of mosaicism and cell‐ and region‐specific differences in gene and protein expression levels between individuals warrants detailed elucidation. It is likely, that the sum of these research efforts will lead to finding the cure of these devastating diseases.

## Conflict of interest

None.
